# Deciphering the Relationship Between Circulating Metabolites and Osteoarthritis: A Comprehensive Genetic Correlation and Mendelian Randomization Studies

**DOI:** 10.7150/ijms.111848

**Published:** 2025-05-16

**Authors:** Guang Yang, Wenqing Xie, Hengzhen Li, Wenhao Lu, Ying Liang, Yusheng Li, Yaping Wang, Wenfeng Xiao

**Affiliations:** 1 Department of Orthopedics, Xiangya Hospital, Central South University, Changsha 410008, Hunan, China.; 2 Molecular Nutrition Branch, National Engineering Research Center of Rice and By-product Deep Processing, College of Food Science and Engineering, Central South University of Forestry and Technology, Changsha 410008, Hunan, China.; 3 National Clinical Research Center for Geriatric Disorders, Department of Geriatrics, Xiangya Hospital, Central South University, Changsha 410008, Hunan, China.; 4 Teaching and Research Section of Clinical Nursing, Xiangya Hospital of Central South University, Changsha 410008, Hunan, China

**Keywords:** Circulating metabolites, OA, MR, LDSC, Genetic Correlation

## Abstract

The causal impact of blood metabolites on OA has yet to be definitively established, further studies are needed to explore the specific roles of metabolites in OA. This is a genetic correlation and two-sample bidirectional mendelian randomization study. GWAS summary data of metabolites and OA were extracted from large-scale GWAS study based on Europeans and Asians. LDSC was conducted to estimate the genetic correlations between 233 circulating metabolites and 11 OA phenotypes, MR was then performed to explore the casual association. 41.20% of the metabolic traits showed genetic correlation with All OA, 15.88% with Knee/Hip OA, 51.50% with Knee OA, and 52.79% with Spine OA. No significant genetic correlations were detected between the metabolic traits and other OA phenotypes. Lactate levels was associated with increased odds of All OA (OR: 1.1558, P<0.001), Hip OA (OR: 1.1446, P=0.004), Knee/Hip OA (OR: 1.1820, P<0.001), Knee OA (OR: 1.1375, P=0.001), Spine OA (OR: 1.3179, P<0.001), THR (OR: 1.5290, P<0.001), and TJR (OR: 1.2827, P<0.001), except for Thumb OA (OR: 0.9429, P<0.001). Ratio of conjugated linoleic acid to total fatty acids was associated 6 OA phenotypes: Hip OA (OR: 0.9522, P=0.035), Knee/Hip OA (OR: 1.0890, P<0.001), Knee OA (OR: 1.1429, P<0.001), THR (OR: 1.3800, P<0.001), TJR (OR: 1.3102, P<0.001), and TKR (OR: 1.2555, P<0.001). Glycerol levels exhibited significant MR associations with four OA phenotypes: Finger OA (OR: 0.6669, P<0.001), Hand OA (OR: 0.8682, P=0.011), Hip OA (OR: 0.9395, P<0.001), and Knee OA (OR: 1.1409, P=0.036). This study underscores genetic and causal connections between specific metabolites and OA. These findings could inform future therapeutic metabolic pathways involved in OA.

## Introduction

Osteoarthritis (OA) is a degenerative disease primarily characterized by joint pain and limited mobility, caused by various factors leading to the fibrosis, fissures, ulceration, and loss of joint cartilage 1. The etiology of OA remains unclear, but its occurrence is associated with age, obesity, inflammation, trauma, and genetic factors 2. The disease manifests pathologically through the degenerative destruction of joint cartilage, sclerosis or cystic changes in the subchondral bone, osteophyte formation at joint edges, synovial changes, and joint capsule contraction 3-5. OA commonly affects the knees, hips, hands, ankles, and spine (including cervical and lumbar regions). Globally, the knee is the most commonly affected site, followed by the hands, other sites, and hips, accounting for approximately 60.6%, 23.7%, 10.2%, and 5.5% of all cases in 2019, respectively 6. Recent data indicate that in 2020, approximately 595 million people worldwide suffered from OA, equivalent to 7.6% of the global population. For individuals over the age of 70, OA has become the seventh leading cause of years lived with disability (YLDs), up from sixth in 1990^7^. It is projected that by 2050, there will be 642 million people with knee OA, 279 million with hand OA, 62.6 million with hip OA, and 118 million with other types of OA 7. Due to global population growth and increased life expectancy, OA, with its high prevalence and associated disability, has become a significant public health challenge. This condition is widespread worldwide and is expected to exert an increasingly profound impact as the population ages.

Metabolism is essential for the functionality of cartilage and synovial joints, and prior research has highlighted a critical role for metabolic processes in inflammatory joint diseases, notably OA 8-10. OA is increasingly recognized as a metabolic-associated disorder, not only because it frequently co-occurs with various metabolic dysfunctions but also due to ongoing discoveries in metabolomics that have identified specific metabolites and metabolic pathways associated with OA 11, 12. Aberrant immune metabolism may represent a key characteristic across many OA phenotypes 13. Recent studies in metabolomics have pinpointed a range of circulating biomarkers in both humans and animal models, including amino acids, carbohydrates, and lipids 14-16. These insights provide new avenues for exploring complex biological metabolic networks and their links to disease states. However, the causal impact of blood metabolites on OA has yet to be definitively established, owing to limitations in sample sizes and potential confounding factors.

Randomized Controlled Trials (RCTs) are considered the gold standard for causal inference in epidemiological studies. However, they are often difficult to conduct due to ethical constraints and high costs 17, 18. Consequently, observational studies are frequently utilized for initial etiological investigations due to their simpler design and easier implementation. Nonetheless, these studies are limited in their capacity for causal inference due to potential confounders and issues of reverse causality 19. Recent advancements in Mendelian randomization (MR) provide a robust method to overcome these limitations 20, 21. MR, a type of instrumental variable analysis, is employed to test causal hypotheses in observational data 22. This approach uses genetic variants as proxies for exposure factors, enabling random grouping and the collection of summary statistics on the associations between these variants and phenotypic outcomes in large populations. By estimating the strength of these associations through genetic epidemiological models, MR minimizes the influence of confounding factors 23. Linkage Disequilibrium Score Regression (LDSC) is an efficient tool for analyzing genetic correlations 24. LDSC differentiates the inflation in statistics due to polygenic effects from confounding due to population stratification or other factors, thereby facilitating the estimation of heritability and genetic correlations between traits based on single nucleotide polymorphisms (SNPs). Previous MR studies investigating the links between metabolites and OA did not include genetic correlation analysis and relied on small sample sizes, which constrained the applicability and statistical robustness of their findings 25. Given this, we aim to conduct more stringent MR analyses using larger, more recent datasets to enhance the reliability and interpretive strength of our research findings.

In this study, we utilized MR to explore the genetic correlations and causal relationships between OA and circulating metabolic biomarkers, drawing upon extensive genome-wide association study data. Our analysis encompassed 233 metabolites and 11 OA phenotypes. By integrating both single-trait and pairwise LDSC, along with bidirectional two-sample MR, this research not only identifies crucial metabolites associated with the risk of OA but also probes their potential mechanisms and interactions. This could provide a scientific foundation for the development of novel preventative and therapeutic strategies, thereby making a positive impact on public health.

## Methods

### Data source and phenotype definition

#### Circulating metabolites exposures

Circulating metabolites were applied as exposures of MR analysis in this study. GWAS summary of 233 circulating metabolic traits was extracted from a large-scale genome-wide association study contains 136,016 participants from 33 cohorts 26. Most of the cohorts consisted of individuals of European ancestry (6 Finnish and 21 non-Finnish), and six cohorts had individuals of Asian ancestry. Circulating metabolic biomarkers was all quantified by nuclear magnetic resonance spectroscopy using NMR metabolomics platform 27. The NMR metabolomics platform provides data of lipoprotein subclasses and their lipid concentrations and compositions, including apoAI and apoB, cholesterol and triglyceride measures, albumin, various fatty acids and low-molecular-weight metabolites.

#### OA outcomes

11 OA phenotypes were selected as outcomes of MR analysis in this study. GWAS summary of 11 OA phenotypes was obtained from large sample genome-wide association study across 826,690 individuals (177,517 with OA) 28. Ethnicity including European (UK, Dutch, Icelandic, Estonian, Greece, European Americans) and Asian (Chinese and Japanese). OA was defined by either a) self-reported osteoarthritis, b) clinical diagnosed, c) ICD10 codes, d) radiographic as defined by the TREAT-OA consortium 29, depending on the data available in the cohort. 11 osteoarthritis phenotypes were descripted as follows: OA at any site (All OA), OA of the hip and/or knee (Knee/Hip OA), OA of the knee (Knee OA), OA of the hip (Hip OA), total joint replacement (TJR), total knee replacement (TKR), total hip replacement (THR), OA of the hand (Hand OA), OA of the finger (Finger OA), OA of the thumb (Thumb OA) and OA of the spine (Spine OA).

### Statistics

#### Linkage disequilibrium score regression analysis

Linkage disequilibrium score regression (LDSC) was performed to estimate genome-wide genetic correlations between exposure and outcomes traits 30. Single-trait LDSC was first used to estimate SNP-based heritability, mean χ2, genome inflation factor (λ_GC_), and the intercept for each GWAS summary statistic. Polygenicity and confounding due to population stratification or cryptic relatedness can be assessed with λ_GC_ and intercept. Pairwise LDSC was conducted to estimate the genomic genetic correlations among the circulating metabolic traits and OA using the pre-computed LD scores of European ancestries from the 1000 Genomes Project Phase 3 (https://alkesgroup.broadinstitute.org/LDSCORE/). Benjamini-Hochberg procedure implemented in R 3.5.3 was used to obtain adjusted p values. P < 0.05 was considered as statistically significant in genetic correlation and MR analyses.

#### Two-sample bidirectional MR analysis

**Figure 1:**
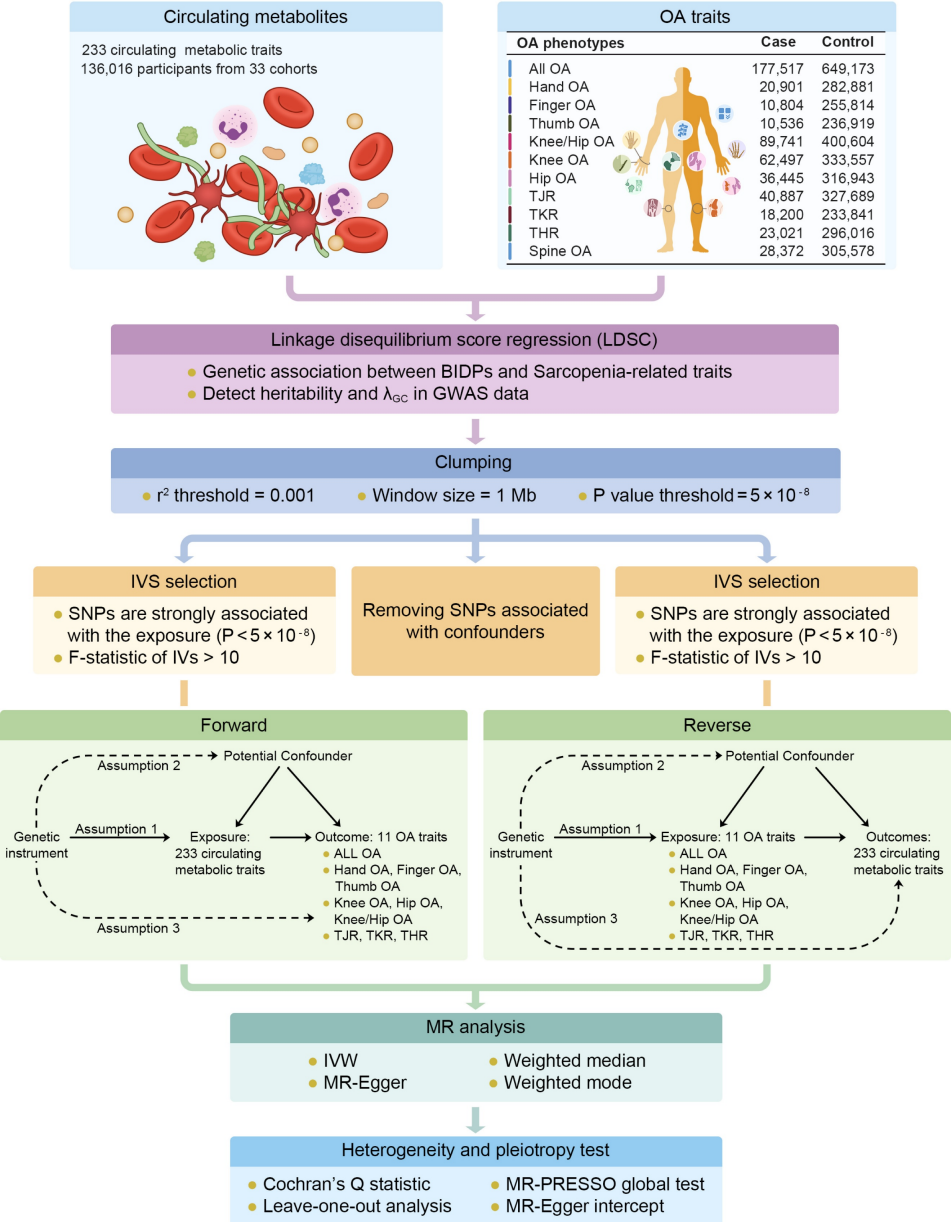
Workflow of genetic correlation and two-sample bidirectional mendelian randomization. Assumption 1: the instrumental variables must be strongly associated with the exposures; Assumption 2: the instrumental variables must be independent of the potential confounders of the association between the exposure and outcome; Assumption 3: the instrumental variables should not be associated with the outcomes directly.

This is a 2-sample bidirectional Mendelian randomization using genetic variants to mimic the effect of circulating metabolic traits on OA traits. The analysis of bidirectional MR is divided into several parts including GWAS data extraction, selection of instrumental variables (threshold selection, clumping selection, pleiotropy selection, F-value selection), forward MR analysis, reverse MR analysis, etc. To ensure the validity of our Mendelian randomization (MR) analysis, we adhered to three core assumptions: Assumption 1: the instrumental variables must be strongly associated with the exposures; Assumption 2: the instrumental variables must be independent of the potential confounders of the association between the exposure and outcome; Assumption 3: the instrumental variables should not be associated with the outcomes directly (Figure 1). For Assumption 1, SNPs with P < 5 × 10^-8^ and F statistic > 10 were selected as instrumental variables (IVs) for MR analysis. In addition, a clumping process (r2 > 0.001, clumping distance = 10,000 kb) was conducted to assess the LD between the included SNPs. For Assumption 2, PhenoScanner [31]and PhenoScanner V2^32^ was used to exclude SNPs strongly (P < 5 × 10^-8^) associated with confounding factors (Age, Sex, BMI, Smoking, Alcohol Consumption, Physical Activity, Diet, Thyroid Dysfunction, and Bone Mineral Density). For Assumption 3, IVs that are significantly associated with the outcome phenotype (P < 5 × 10^-8^) were excluded.

To conduct MR estimates, the random-effects model inverse-variance weighted (IVW) method was applied as the primary statistical analysis approach 33. Random-effect IVW were conducted to reduce bias when heterogeneity exists 34. The weighted median method, MR Egger regression method, weighted mode method and simple mode method were also performed as sensitivity analyses. MR-Egger intercept test was performed as an indicator of directional pleiotropy (P < 0.05 was considered statistically significant). MR-PRESSO test was also used to evaluate overall horizontal pleiotropy and detect SNP outliers. Cochran's Q statistic was used to check the heterogeneity among SNPs. The leave-one-out method was employed to evaluate whether single SNP could exert bias to the IVW estimate. The R packages “TwoSampleMR” (version 0.6.4) were used to conduct Mendelian randomization. Benjamini-Hochberg procedure implemented in R (version 4.3.2) was used to obtain adjusted p values, *P* < 0.05 was considered as statistically significant in genetic correlation and MR analyses.

## Results

### Single-trait and Pairwise LDSC result

**Figure 2:**
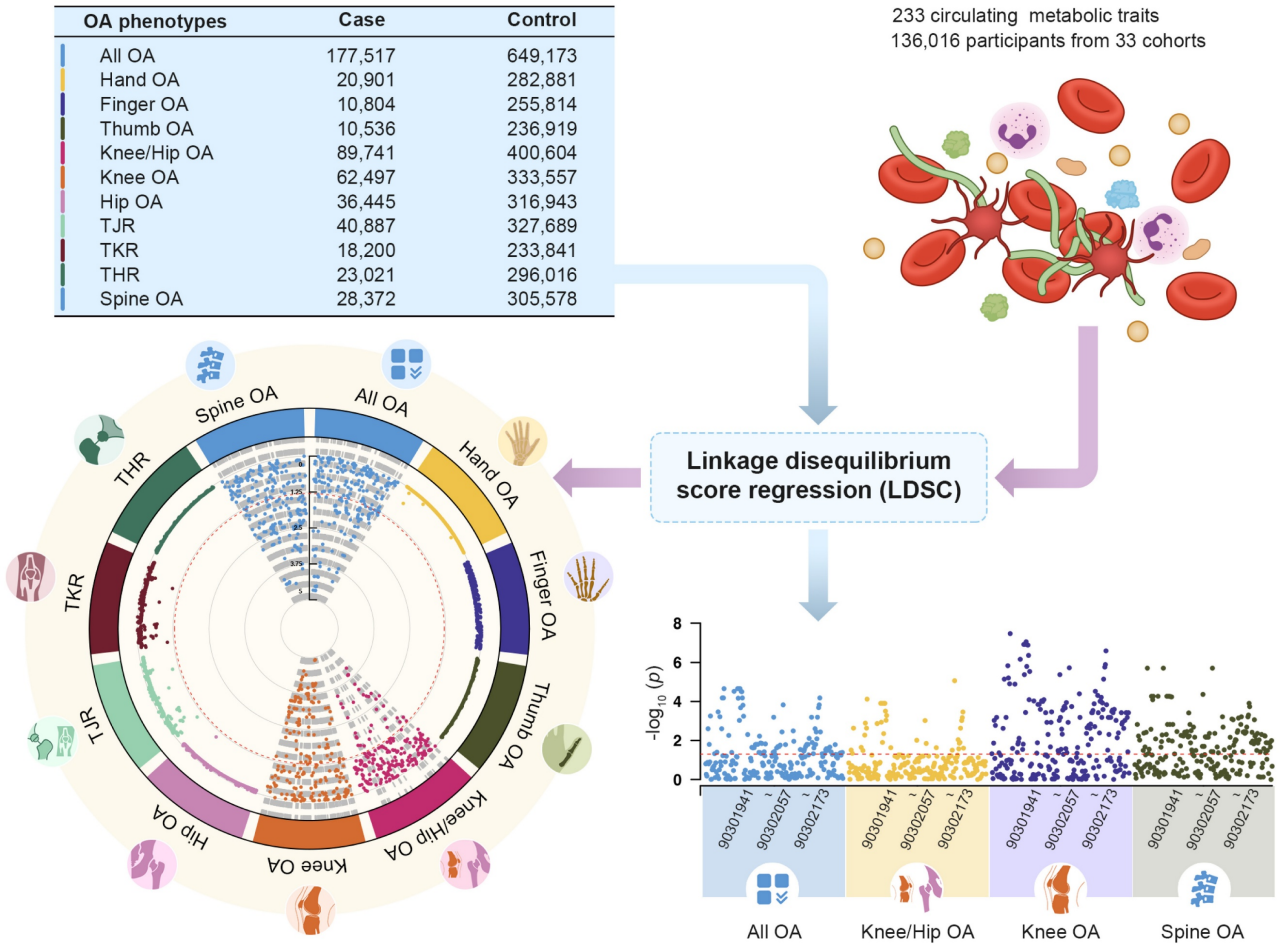
The genetic correlation between 233 circulating metabolic traits and 11 OA phenotypes.

Single-trait LDSC analysis showed that the average heritability of 233 metabolic traits calculated based on GWAS summary data was 0.0996, with a minimum value of 0.0147 and a maximum value of 0.1644. The average genomic control inflation factor (λ_GC_) was 1.1641, ranging from a minimum of 1.0405 (Acetate levels) to a maximum of 1.2498 (Triglycerides to total lipids ratio in chylomicrons and extremely large VLDL) (Table S1). For 11 OA phenotypes, the average heritability was 0.0146, ranging from a minimum of 0.0035 to a maximum of 0.0245. The average λ_GC_ of 11 OA traits was 1.1808, ranging from a minimum of 1.0557 (Finger OA) to a maximum of 1.4037 (Knee/Hip OA) (Table S2). Pairwise LDSC analysis revealed that 41.20% of the metabolic traits showed a genetic correlation with All OA, 15.88% with Knee/Hip OA, 51.50% with Knee OA, and 52.79% with Spine OA (Figure 2). There were no significant genetic correlations observed between the metabolic traits and other OA phenotypes (Table S3).

### Association between Circulating Metabolites and OA in loading extremity joint

**Figure 3:**
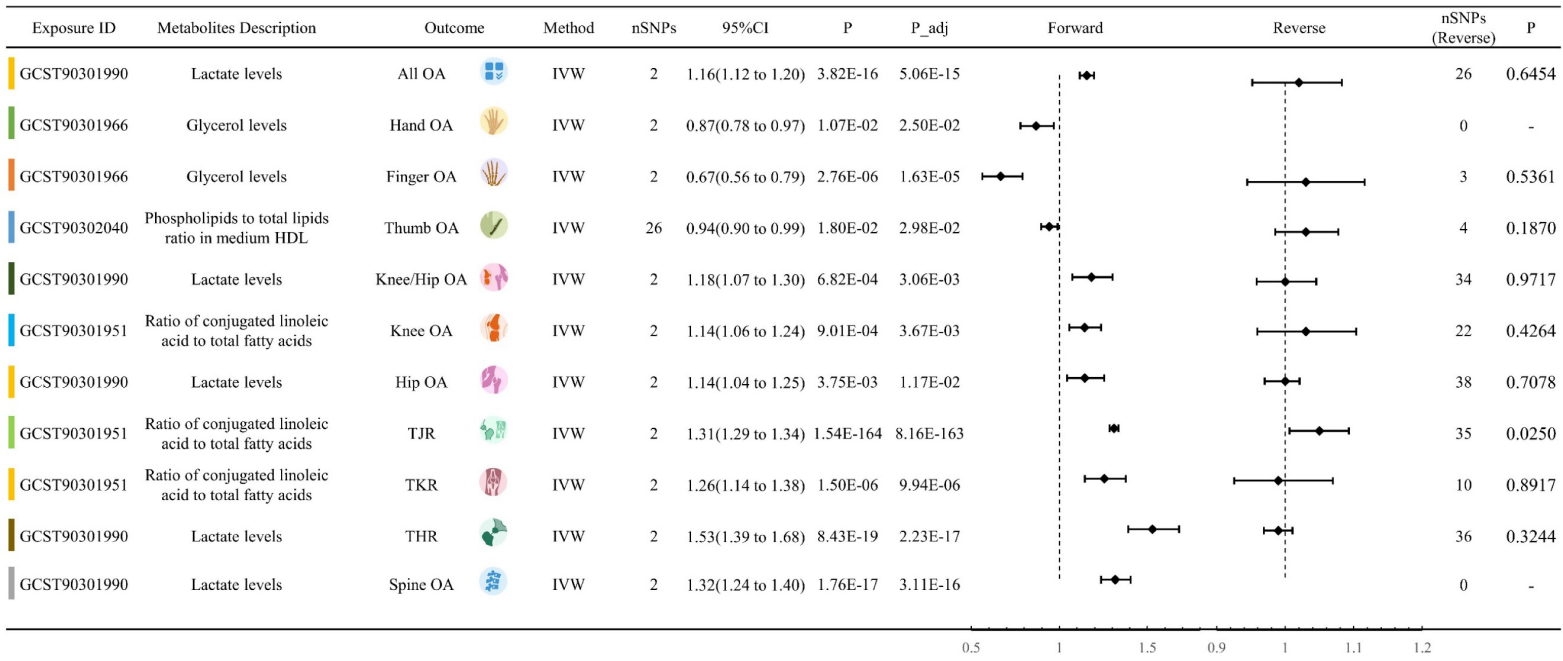
Representation of the metabolites with the highest absolute value of OR-1 among 11 OA trait.

8 circulating metabolites traits showed significant forward MR relationships with Knee/Hip OA. These traits were Ratio of conjugated linoleic acid to total fatty acids, Ratio of diacylglycerol to triglycerides, Isoleucine levels, Lactate levels, Phospholipids in large VLDL, Concentration of medium VLDL particles, Total cholesterol to total lipids ratio in very large HDL, and Phospholipids to total lipids ratio in very large HDL. Among them, Lactate levels had the highest absolute values of OR-1(OR: 1.1820, 95% CI: 1.0733 to 1.3017, P = 0.003) (Figure 3). 3 circulating metabolites traits showed significant associations with Knee OA, namely Ratio of conjugated linoleic acid to total fatty acids, Glycerol levels, and Lactate levels. Lactate levels exhibited the highest absolute values of OR-1 (OR: 1.1375, 95% CI: 1.0523 to 1.2296, P = 0.004). 7 circulating metabolites traits showed significant associations with Hip OA, including Ratio of conjugated linoleic acid to total fatty acids, Glycerol levels, Total cholesterol levels in HDL, Lactate levels, Total cholesterol to total lipids ratio in small LDL, Cholesteryl esters to total lipids ratio in very large HDL, and Phospholipids in very large HDL, Lactate levels obtained the highest absolute values of OR-1 (OR: 1.1446, 95% CI: 1.0447 to 1.2540, P = 0.012). In addition, 3 circulating metabolites traits were associated with THR, eight with TJR, and three with TKR (Table S4). Reverse MR analysis indicated a significant MR effect of Knee OA on Lactate levels (OR: 1.0646, P = 0.008). THR and TJR were both significantly associated with Ratio of conjugated linoleic acid to total fatty acids, with OR values of 1.0294 and 1.0485, respectively.

### Association between Circulating Metabolites and OA in non-loading extremity joint

4 circulating metabolites traits showed significant forward MR relationships with Hand OA. These traits were Glycerol levels, Phospholipids to total lipids ratio in medium HDL, Cholesterol esters in very large HDL, and Triglycerides in very large HDL. Among them, Triglycerides in very large HDL had the highest OR-1 value (OR: 0.9730, 95% CI: 0.9492 to 0.9974, P = 0.039). 5 circulating metabolites traits showed significant MR relationships with Finger OA. These traits were Acetate levels, Ratio of 22:6 docosahexaenoic acid to total fatty acids, Glycerol levels, Free cholesterol in large HDL, and Pyruvate levels. Among them, Acetate levels had the highest OR-1 value (OR: 0.8266, 95% CI: 0.6914 to 0.9882, P = 0.040). 4 circulating metabolites showed significant associations with Thumb OA. These traits were Free cholesterol to total lipids ratio in IDL, Lactate levels, Total cholesterol to total lipids ratio in large HDL, and Phospholipids to total lipids ratio in medium HDL. Among them, Phospholipids to total lipids ratio in medium HDL had the highest absolute values of OR-1 (OR: 0.9421, 95% CI: 0.8966 to 0.9898, P = 0.030) (Table S4). Reverse MR analysis did not detect statistically significant results (Table S5).

### Association between Circulating Metabolites and OA in spine

6 circulating metabolites traits showed significant forward MR relationships with Spine OA. These traits were 3-Hydroxybutyrate levels, Lactate levels, Ratio of polyunsaturated fatty acids to total fatty acids, Pyruvate levels, Total cholesterol to total lipids ratio in very large HDL, and Phospholipids to total lipids ratio in very large HDL. Among them, Lactate levels had the highest absolute values of OR-1 (OR: 1.3179, 95% CI: 1.2367 to 1.4044, P < 0.001). Both Pyruvate levels and Total cholesterol to total lipids ratio in very large HDL had OR effect values less than 1 for Spine OA (Table S4). Reverse MR analysis did not detect statistically significant results.

### Circulating Metabolites exerting multi-dimensional effects on OA traits

**Figure 4:**
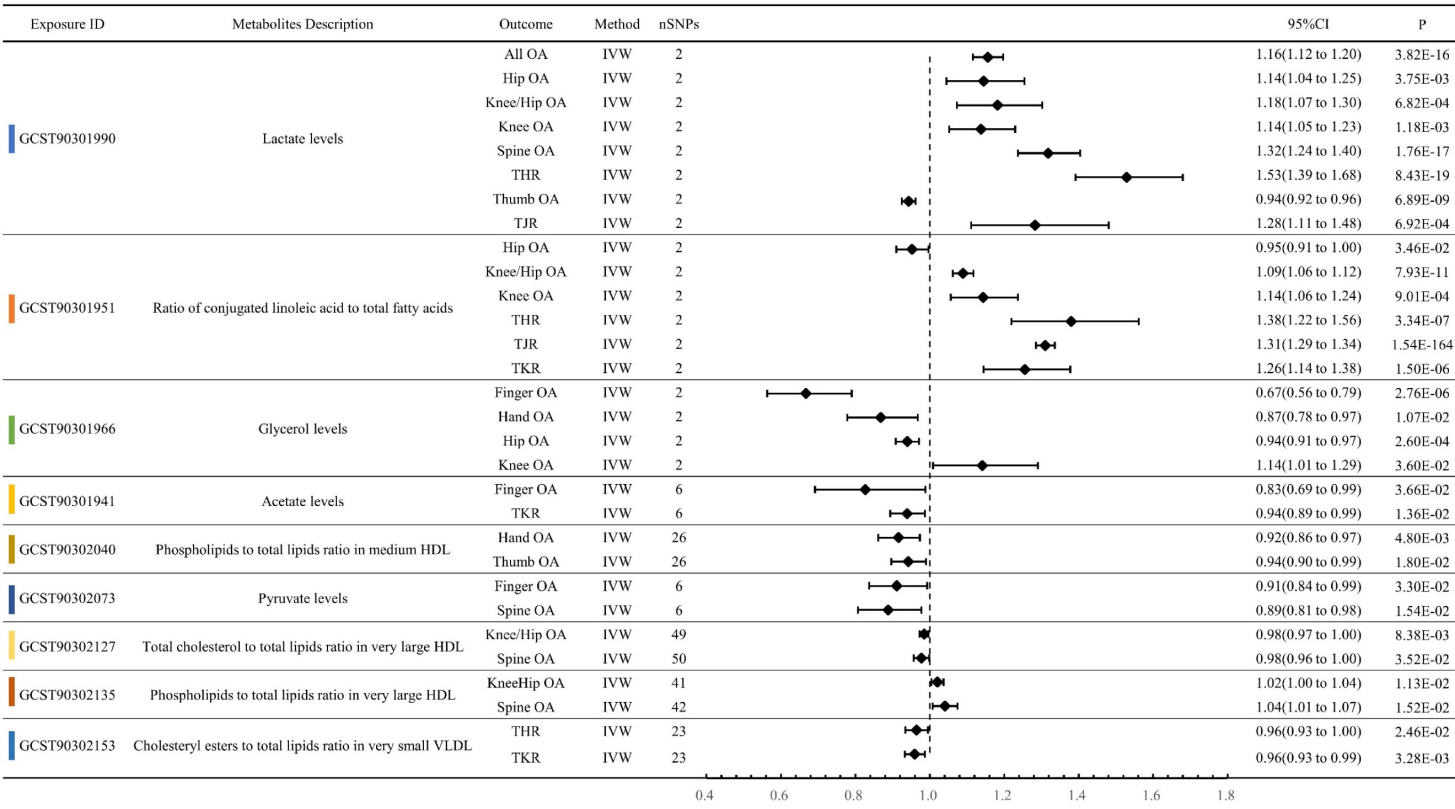
Forest plot of circulating metabolites exerting multi-dimensional effects on OA traits.

Lactate levels exhibited a significant forward MR relationship with eight OA phenotypes. Except for Thumb OA (OR: 0.9429, P < 0.001), the OR values for the other OA phenotypes were greater than 1. Specifically, they were All OA (OR: 1.1558, P < 0.001), Hip OA (OR: 1.1446, P = 0.004), Knee/Hip OA (OR: 1.1820, P < 0.001), Knee OA (OR: 1.1375, P = 0.001), Spine OA (OR: 1.3179, P < 0.001), THR (OR: 1.5290, P < 0.001), and TJR (OR: 1.2827, P < 0.001). Ratio of conjugated linoleic acid to total fatty acids also showed significant MR relationships with 6 OA phenotypes: Hip OA (OR: 0.9522, P = 0.035), Knee/Hip OA (OR: 1.0890, P < 0.001), Knee OA (OR: 1.1429, P < 0.001), THR (OR: 1.3800, P < 0.001), TJR (OR: 1.3102, P < 0.001), and TKR (OR: 1.2555, P < 0.001). Moreover, Glycerol levels exhibited significant MR associations with four OA phenotypes: Finger OA (OR: 0.6669, P < 0.001), Hand OA (OR: 0.8682, P = 0.011), Hip OA (OR: 0.9395, P < 0.001), and Knee OA (OR: 1.1409, P = 0.036) (Figure 4, Table S6).

### Sensitivity Analysis

In 11 OA phenotypes, the metabolite with the largest absolute value of OR-1 is shown in Figure 3. The MR results of these phenotypes all exhibit statistical significance in the IVW method, but do not reach significance in other methods. Except for the effect of Phospholipids to total lipids ratio in medium HDL on ThumbOA, which also shows significance in Weighted median, Weighted mode, and Simple mode (Table S7). In the metabolites illustrated in Figure 4, most metabolites show statistical significance in the IVW method, but not in other MR methods. Except for Phospholipids to total lipids ratio in medium HDL, Pyruvate levels, Total cholesterol to total lipids ratio in very large HDL, Phospholipids to total lipids ratio in very large HDL, and Cholesteryl esters to total lipids ratio in very small VLDL, the significance of these metabolites is statistically significant in four or more MR methods. ALL sensitivity analysis result can be referenced in Table S8.

## Discussion

This study employed MR to investigate the potential genetic correlations and causal relationships between circulating metabolic biomarkers and OA. OA is a prevalent degenerative joint disease that significantly impacts the quality of life for millions globally. Despite the incomplete understanding of its precise etiology, a growing body of epidemiological evidence indicates an association between metabolic disturbances and OA, particularly in younger demographics 35. These disturbances include conditions such as obesity, hypertension, dyslipidemia, hyperglycemia, and insulin resistance, all of which can accelerate the onset and progression of OA across both weight-bearing and non-weight-bearing joints 36. Our systematic analysis of 233 metabolites and 11 OA phenotypes revealed notable genetic and causal connections between various metabolites and distinct OA phenotypes. Results from paired LDSC analysis indicated significant genetic correlations between numerous metabolites and specific OA manifestations, including knee and spinal OA. Furthermore, our comprehensive MR analysis assessed the relationships between circulating metabolites and various types of OA, encompassing weight-bearing joints (knees and hips), non-weight-bearing joints (hands and fingers), and spinal OA. Our findings demonstrate that certain metabolites, such as lactate levels, the ratio of conjugated linoleic acid to total fatty acids, and glycerol levels, exhibit significant positive MR associations with various OA phenotypes. These results suggest that these metabolites might influence the progression of OA through specific biological pathways, offering insights that could inform future preventative and therapeutic strategies for OA.

This study identified a significant positive association between lactate levels and eight OA phenotypes through MR analysis. With the exception of Thumb OA (OR: 0.9429), lactate levels showed a consistent direction of effect for the remaining seven OA phenotypes, specifically indicating positive correlations with All OA, Hip OA, Knee/Hip OA, Knee OA, Spine OA, THR, and TJR. These findings suggest that elevated lactate levels are strongly linked to an increased risk of these OA phenotypes, underscoring the pathological significance of lactate as a metabolic factor in OA. Traditionally viewed as a metabolic waste product of glycolysis, recent studies have revealed that lactate serves not merely as an intermediary metabolite but also as a versatile immunomodulator that regulates inflammatory responses 37, 38. In the synovium of chronic arthritis, lactate influences immune cell function through multiple mechanisms, including migration and cytokine production 39. Lactate triggers a "stop migration" signal in T cells through the lactate transporters SLC5A12 in CD4+ T cells and SLC16A1 in CD8+ T cells, promoting their accumulation at inflammation sites 40, 41. This lactate-mediated suppression of T cell mobility in inflamed tissues coincides with a reduction in glycolysis. Sodium lactate inhibits several glycolytic enzymes and decreases glucose flux expression in CD4+ T cells, leading to T cell accumulation at the site of inflammation. This process extends the duration of chronic inflammation by increasing the production of inflammatory cytokines and reducing cell lysis 42, 43. OA is a chronic inflammatory joint disease, and lactate is known to exacerbate its progression through previously described pathways. Recent research has underscored the significant role of lactate in the pathogenesis of OA, revealing elevated levels of lactate in patients and linking these levels to various metabolic indicators 44. Studies have demonstrated that lactate activates the HCAR1/PI3K signaling pathway, which upregulates the expression of NADPH oxidase 4 (NOX4). This upregulation leads to an increased production of reactive oxygen species (ROS), resulting in chondrocyte dysfunction. Furthermore, lactate is shown to promote the expression of degradative enzymes, reduce the synthesis of type II collagen, and induce the secretion of inflammatory factors, as well as chondrocyte hypertrophy and senescence. The application of the NOX4 inhibitor GLX351322 or the ROS scavenger N-acetylcysteine (NAC) has been found to mitigate the damage to chondrocytes induced by lactate 44. Additionally, intra-articular injections of oxamate (an LDHA inhibitor that reduces the conversion of pyruvate to lactate) effectively decrease the expression of glycolysis-related proteins in a rat model of OA induced by anterior cruciate ligament transection (ACLT). This treatment significantly reduces chondrocyte apoptosis and alleviates pain and inflammation, while also inhibiting cartilage degeneration 45. Both past studies and our findings highlight the role of lactate as a metabolic factor in OA progression, providing a theoretical foundation and potential targets for future metabolic treatment strategies.

Moreover, this study's findings reveal that the ratio of conjugated linoleic acid (CLA) to total fatty acids exhibits distinct association patterns across various OA phenotypes. Specifically, this ratio displays a protective effect in Hip OA (OR: 0.9522), suggesting that a higher proportion of CLA relative to total fatty acids may decrease the risk of Hip OA. Conversely, in Knee/Hip OA (OR: 1.0890), Knee OA (OR: 1.1429), THR (OR: 1.3800), TJR (OR: 1.3102), and TKR (OR: 1.2555), a higher ratio correlates with an increased disease risk, indicating that an elevated ratio of conjugated linoleic acid to total fatty acids may constitute a risk factor in these phenotypes. CLA, an unsaturated octadecadienoic acid containing conjugated double bonds, encompasses a mix of positional and geometric isomers of linoleic acid 46. It is known for its physiological activities, which include anti-inflammatory, antihypertensive, anticancer, antioxidant, anti-atherosclerosis, and anti-obesity effects 47. Theoretically, CLA includes 56 isomers, with 20 naturally occurring isomers identified through NMR, each exhibiting significantly different physiological functions 48. Among these, the cis-9, trans-11 and trans-10, cis-12 isomers are the most abundant and are noted for their beneficial physiological activities 49-51. CLA is typically recognized for its anti-inflammatory and antioxidant properties 52, 53, which may elucidate its protective role in Hip OA. However, in other OA phenotypes, increased levels of CLA might worsen disease risk through mechanisms such as altered lipid metabolism and heightened inflammatory responses. Particularly in severe OA phenotypes requiring joint replacement, the risk increase associated with high CLA ratios could be linked to its pro-inflammatory or detrimental metabolic effects within the local environment. This dual effect implies that the influence of CLA may vary based on the type and pathological state of OA. Additionally, these varying impacts of CLA could also be attributed to its different isomers 48. Various CLA isomers might have distinct roles in lipid metabolism, inflammation regulation, and immune function, thus affecting the development and progression of OA. Future research should explore in greater depth the specific biological mechanisms of different CLA isomers across various OA phenotypes and assess their potential as therapeutic targets to better comprehend and manipulate their role in OA progression. This molecular-level understanding could help in precisely regulating CLA-related pathways, providing more targeted strategies for OA treatment. In the realm of experimental research, a study conducted by Shen CL and colleagues provides preliminary evidence concerning the potential role of CLA in OA treatment 54. Theirs in vitro experiments assessed the impact of CLA, both alone and in combination with other polyunsaturated fatty acids (PUFAs), on the production of inflammatory mediators in human OA chondrocytes. The study found that CLA significantly decreased the production of prostaglandin E2 and nitric oxide, with the combination of CLA and eicosapentaenoic acid (EPA) proving particularly effective in reducing PGE2 levels. These findings indicate that CLA could play a role in OA treatment by modulating inflammatory mediators. However, given the relatively sparse research on CLA's effects on OA, further investigations are required to confirm these results and to explore the specific mechanisms through which CLA and its isomers act in various types and pathological states of OA. A deeper molecular understanding will facilitate the precise modulation of CLA-related pathways, providing more targeted therapeutic strategies for OA treatment.

This study also uncovered significant correlations between glycerol levels and various OA phenotypes: glycerol levels were inversely associated with both finger OA and hand OA, indicating a potential protective role for glycerol in these non-weight-bearing joints. Additionally, glycerol demonstrated a negative association with hip OA, suggesting it may protect joints subjected to lower mechanical stress. This protective effect may be due to glycerol's role in regulating lipid metabolism and anti-inflammatory pathways, which helps to maintain cell membrane stability and suppress the production of inflammatory mediators, thereby mitigating joint tissue damage and inflammation. Conversely, the positive correlation between glycerol levels and weight-bearing joints such as knee OA suggests a different mechanism might be at play. In such weight-bearing joints, increased levels of glycerol could be associated with disorders in lipid metabolism, leading to heightened local or systemic inflammatory states, thereby promoting the progression of OA. Furthermore, as an energy source, glycerol might cause imbalances in energy metabolism under conditions of excessive joint usage or increased mechanical stress, further aggravating degenerative changes in joint cartilage. These findings suggest potential therapeutic targets for different OA phenotypes. For example, modulating glycerol levels or its metabolic pathways could offer protective strategies for hand and finger joint OA, whereas strategies to decrease glycerol levels or adjust its related metabolic pathways may be beneficial for knee joint OA. Additionally, these results underscore the importance of personalized medicine, that is, developing treatment plans tailored to the specific OA phenotypes and metabolic characteristics of individual patients. These insights should be further explored in future research, particularly through clinical trials that assess the impact of modulating glycerol levels or its metabolic pathways on OA progression, as well as the safety and efficacy of such interventions.

While this study offers new insights into the associations between circulating metabolic biomarkers and OA, it is subject to several limitations. Firstly, the study used data from multiple ethnicities, but genetic differences across populations can impact associations between genetic variants and metabolic traits, potentially introducing bias in MR analysis. Secondly, the MR analysis in this study uses genetic instrumental variables to represent exposure factors. Due to the constraints of the sample size and the additive regression models used, only a small proportion of the variance in exposure factors is explained, making it difficult to detect subtle causal effects among complex traits. Lastly, the effect sizes derived from genetic correlation analyses represent estimates based on the current dataset and model, and should not be considered equivalent to or substitutes for effect sizes obtained from observational clinical studies. A more comprehensive understanding beneficial for clinical practice can only be achieved by integrating genetic correlation analysis with traditional epidemiological studies, real-world research, bibliometric reviews, or meta-analyses.

## Conclusion

This study utilized MR and LDSC methods to identify significant genetic and causal links between circulating metabolites and various OA phenotypes. Lactate levels were positively associated with multiple OA phenotypes, suggesting their role in the processes of OA pathogenesis. The ratio of conjugated linoleic acid to total fatty acids and glycerol levels showed variable effects across different OA types, suggesting their potential as therapeutic targets. These findings emphasize the importance of metabolic profiling in OA management, supporting personalized medicine approaches. Future research should further explore these metabolites' mechanisms and validate the findings across diverse populations and clinical settings.

## Supplementary Material

Supplementary figures and tables.
